# Bilateral Uveal Melanoma: An Insight into Genetic Predisposition in Four New Unrelated Patients and Review of Published Cases

**DOI:** 10.3390/jcm13113035

**Published:** 2024-05-22

**Authors:** Paula Silva-Rodríguez, Manuel Bande, María Pardo, Fernando Domínguez, Lourdes Loidi, María José Blanco-Teijeiro

**Affiliations:** 1 Fundación Pública Galega de Medicina Xenómica (FPGMX), 15706 Santiago de Compostela, Spain; paula.silva.rodiguez@gmail.com (P.S.-R.); lourdes.loidi.fernandez@sergas.es (L.L.); 2 Grupo de Oftalmología Traslacional, Área Oncología, Instituto de Investigación Sanitaria de Santiago de Compostela (IDIS), 15706 Santiago de Compostela, Spain; maria.jose.blanco.teijeiro@sergas.es; 3 Department of Ophthalmology, Clinical University Hospital of Santiago de Compostela, 15706 Santiago de Compostela, Spain; 4 Grupo Obesidómica, Área de Endocrinología, Instituto de Investigación Sanitaria de Santiago de Compostela (IDIS), 15706 Santiago de Compostela, Spain; maruxapardo@hotmail.com; 5 Department of Physiology and Centro de Investigaciones en Medicina Molecular y Enfermedades Crónicas (CiMUS), University of Santiago de Compostela, 15782 Santiago de Compostela, Spain; fernando.dominguez@usc.es

**Keywords:** bilateral uveal melanoma, incidence, genetic predisposition, candidate genes

## Abstract

**Background**: Primary bilateral uveal melanoma (BUM) is an exceptionally rare form of uveal melanoma (UM). This study aimed to explore the potential existence of a genetic predisposition towards the development of BUM. **Methods**: We employed an exome sequencing approach on germline DNA from four unrelated patients diagnosed with BUM, seeking pathogenic or likely pathogenic variants indicative of a genetic predisposition to UM. **Results**: None of the patients exhibited pathogenic variants in the *BAP1* gene. However, loss-of-function (LoF) variants in the *TERF2IP* and *BAX* genes were identified in two of the BUM patients. For patients BUM1 and BUM2, no pathogenic/likely pathogenic variants of significant clinical relevance to BUM were found to warrant inclusion in this report. **Conclusions**: Our findings suggest the presence of yet-to-be-discovered genes that may contribute to UM predisposition, as evidenced by the absence of pathogenic variants in known UM predisposition genes among the four BUM patients studied. The *TERF2IP* and *BAX* genes emerge as noteworthy candidates for further investigation regarding their role in genetic predisposition to UM. Specifically, the potential role of UM as a candidate cancer within the spectrum of cancers linked to pathogenic variants in the *TERF2IP* gene and other genes associated with the *shelterin* complex warrants further examination. Additional functional studies are necessary to support or challenge this hypothesis.

## 1. Introduction

Uveal melanoma (UM) stands as the most prevalent primary intraocular tumor in adults, although it remains a relatively rare malignancy. Typically, UM presents as a unilateral and unifocal condition; however, the occurrence of primary bilateral uveal melanoma (BUM) is exceptionally rare. Our current understanding of primary BUM primarily stems from a limited number of small case series and isolated reports [1,2,3,4,5,6,7,8]. The most extensive study, encompassing the largest collection of BUM cases, synthesized information from forty-six rigorously documented articles published in English between 1950 and 2016. Specifically, this is a population-based study and systematic review conducted using three sources: a pathology database spanning from 1996 to 2016, the Surveillance, Epidemiology, and End Results (SEER)-18 registry covering the period from 1973 to 2013, and published English literature. Altogether, these sources provided a comprehensive compilation of fifty-two cases of BUM [8]. To date, the scientific literature, including databases such as PubMed, has reported no more than seventy cases of BUM (Supplementary Table S1).

The onset of UM is generally sporadic, arising without any precursor lesions. However, it has been associated with various risk factors, including the transformation of a preexisting benign ocular nevus, the presence of ocular melanocytosis, and the development of congenital syndromes that lead to melanocytic lesions and cancer [9,10]. Specifically, bilateral ocular melanocytosis has been implicated in the development of multifocal or bilateral UM forms [4,11,12], suggesting its role as a risk factor for BUM. Yet, not all BUM cases can be explained by pre-existing melanocytic lesions.

The hypothesis of a genetic predisposition to BUM is grounded in the observation that bilateral involvement of paired organs is a hallmark of cancer predisposition syndromes [13,14]. Identifying individuals at risk enables the implementation of cancer preventive strategies, such as prophylactic surgeries or early interventions, to forestall or mitigate the disease’s progression.

In BUM cases, the only gene conclusively linked to its development is *BAP1*, which encodes the Breast Cancer Type 1 Susceptibility (BRCA1)-Associated Protein 1 [7,15]. The paucity of information on the molecular mechanisms underlying BUM suggests the potential involvement of unidentified germline mutations in other susceptibility genes. This notion was proposed by Singh et al. in 1996, preceding their report on two unrelated BUM patients with germline mutations in the *BAP1* gene [1,7].

Pathogenic germline variants in other genes, such as *MBD4*, have been reported in UM patients in relation to the predisposition to develop such tumors [16,17,18]. Additionally, certain case reports have linked UM development to germline mutations in genes like *CDKN2A*, *BRCA1/BRCA2*, *MLH1*, *MSH6*, *FLCN*, and *POT1* [19,20,21,22,23,24]. Recent findings suggest moderate evidence of hereditary predisposition to UM through germline mutations in *MLH1* or *PALB2*, indicating locus heterogeneity for UM predisposition [25]. All those discoveries mark the first indication of other moderate and high-penetrance genes that have yet to be identified.

In this context, our study aims to explore the potential existence of predisposing germline mutations involved in the development of BUM. We conducted exome sequencing on germline DNA samples from four unrelated Caucasian patients diagnosed with BUM.

## 2. Materials and Methods

### 2.1. Patient Cohort

This study included four unrelated patients diagnosed with BUM, under the care of the ophthalmology department at the Santiago de Compostela hospital complex. Eligibility criteria included a lack of familial history of UM or any cancer at the onset of the study, a negative result for metastatic screening, and a confirmed diagnosis of BUM through comprehensive ophthalmic examination and full eye ultrasonography. Written informed consent was secured from all participants, and the study received approval from the local Ethics Committee, in accordance with the Declaration of Helsinki principles (Registration Code: 2009/128, Date for approval: 22 December 2022).

### 2.2. Exome Sequencing and Genetic Testing

Exome sequencing was conducted on germline DNA extracted from peripheral blood cells of the four BUM patients. The DNA extraction was performed using the CHEMAGEN robot (Chemagen Biopolymer-Technologie AG, Baesweiler, Germany). For three patients (BUM1-3), sequencing strategy utilized the SureSelect Focused Exome (Agilent Technologies, Santa Clara, CA, USA) and was run on an IonTorrent PGM sequencing platform (Thermo Fisher Scientific, Waltham, MA, USA), while the last diagnosed patient (BUM4) underwent sequencing with the xGen Exome Research Panel v2 on the Nextseq 500 (Illumina, San Diego, CA, USA). BUM1, BUM2, and BUM3 were also screened for germline mutations in the *TERF2IP* gene using a specific ad hoc gene panel. Sequencing analysis referenced the human genome sequence UCSC build hg19 (NCBI build 37). Detected variants were visualized using the Integrative Genomics Viewer (IGV) software (Broad Institute, Cambridge, MA, USA) (https://www.broadinstitute.org/scientific-community/software/integrative-genomics-viewer), achieving 96% coverage with an average sequencing depth of 146X for the xGen Exome Research Panel v2 and 177X with 94% coverage for the SureSelect Focused Exome. Variant classification adhered to the American College of Medical Genetics (ACMG) guidelines [26]. A multi-gene targeting strategy was used to search for recurrent somatic mutations in UM in formalin-fixed paraffin-embedded tumor samples (FFPE), using AmpliSeq technology (Thermo Fisher, Waltham, MA, USA) as described in our previously published study [27].

### 2.3. Variant Prioritization and Candidate Gene Selection

Variants were initially filtered based on frequency, focusing on rare variants with a Minor Allele Frequency (MAF) of ≤1% or unreported in the Non-Finnish European population (NFE) in the Genome Aggregation Database v2.1.1 (gnomAD; [28]). Pathogenic or likely pathogenic variants were defined as null variants (stop-gain, start loss, frameshift, canonical splice sites ±2 bp from exons) and missense variants reported as pathogenic/likely pathogenic in ClinVar [29] or in the Human Gene Mutation Database (HGMD) [30]. Emphasis was placed on well-known cancer genes [31], other putative cancer-related genes, and DNA repair genes [32] potentially associated with cancer predisposition (Supplementary Table S2).

Gene prioritization was based on a thorough literature review to identify existing functional studies and other bibliographic evidence. Information about the described genes was primarily obtained from the GeneCards repository https://www.genecards.org (accessed on 1 January 2021), the Human Phenotype Ontology Browser (HPO; [33]), the Online Mendelian Inheritance in Man (OMIM; [34]), the Human Protein Atlas available at http://www.proteinatlas.org (accessed on 1 January 2021) [35] and the UniProt database https://www.uniprot.org/ (accessed on 1 January 2021) [36].

## 3. Results

Characteristics of the Patient Cohort: None of the four BUM patients had any prior history of cancer or familial records of UM or other cancer types. Patient BUM4 developed primary breast cancer during the course of this study. The etiology of BUM, excluding metastases from the contralateral eye or other primary cancers, was confirmed for all four patients. Prognostic studies using Multiplex ligation-dependent probe amplification (MLPA) test were not possible due to either the absence of a tumor sample from the first diagnosed melanoma or patient refusal of treatment for the second melanoma.

**Figure 1:**
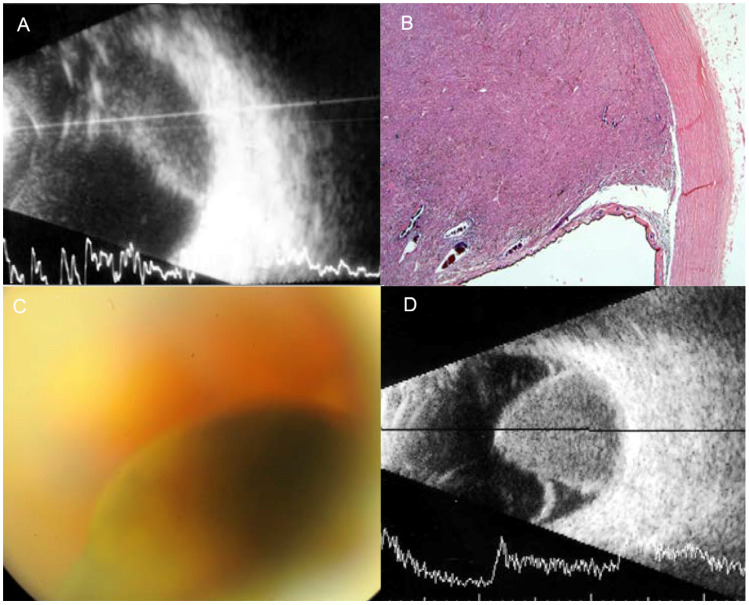
Imaging test results for the diagnosis of BUM in patient BUM1. (**A**) Ocular ultrasound showing the presence of the first choroidal mass in the patient’s left eye. (**B**) Histological slice of the UM, obtained after enucleation of the LE. (**C**) Fundus of the right eye, showing the UM diagnosed in the patient in 2016. (**D**) Ocular ultrasound scan of the patient’s right eye.

Patient BUM1: This individual, an 80-year-old healthy Caucasian man, developed BUM over a sixteen-year period. In 1997, diagnostic examinations including ophthalmoscopy and ultrasonography identified a pigmented choroidal mass located in the temporal quadrant of the left eye (LE), consistent with UM. Due to the substantial base dimension of the mass, exceeding 16 mm, and its involvement with the ciliary body, the medical team opted for enucleation of the LE. Pathological evaluation post-surgery confirmed the presence of fusiform B UM, classified as stage T3b. Additionally, regular monitoring was recommended for a choroidal nevus detected in the right eye (RE). In 2013, a sudden decrease in visual acuity led to the detection of UM (T2, AJCC stage 8th [37]) in the RE’s inferior quadrants (Figure 1). After receiving brachytherapy in the RE, the patient succumbed to liver metastases a year later. Somatic genetic analysis on an FFPE sample from the first tumor identified the recurrent p.(Gln209Leu) mutation in the *GNA11* gene. Unfortunately, the DNA quality was too poor for further prognostic genetic testing via MLPA.

**Figure 2:**
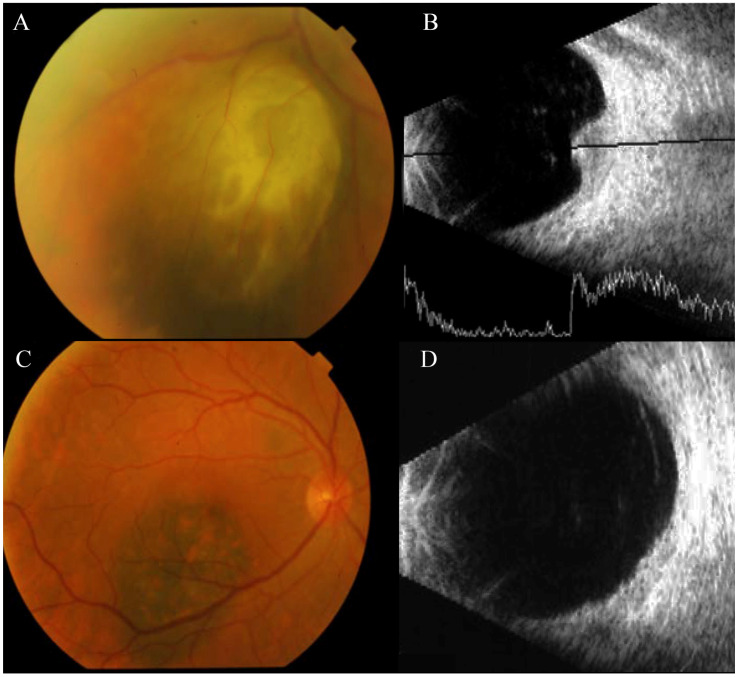
Imaging tests corresponding to the diagnosis of BUM in patient BUM2. (**A**) Fundus of the eye showing the UM detected in the patient’s left eye. (**B**) Ocular ultrasound scan carried out on the left eye showing the presence of UM located in the lower nasal quadrant. (**C**) Fundus of the patient’s right eye, showing a mass located below the fovea compatible with the diagnosis of a small melanoma. (**D**) Ocular ultrasound scan of the RE of the patient, showing a choroidal mass compatible with a melanoma.

Patient BUM2: A 56-year-old Caucasian man experienced a progressive, mild decrease in visual acuity in his LE and floaters in both eyes. Retinography and ocular ultrasonography identified a pigmented choroidal mass in the lower nasal quadrant of the LE, consistent with a diagnosis of UM at stage T2. Concurrently, a small melanocytic lesion was observed in the RE, situated below the fovea. Despite being a very flat lesion, it exhibited typical features of UM at stage T1, such as the presence of orange pigment and rapid growth (Figure 2).

In light of these findings, the patient underwent comprehensive screening for metastatic lesions, which returned negative results. The specialists recommended a conservative approach, opting for I125 brachytherapy for both eyes. However, the patient declined treatment for the melanoma identified in the RE and proceeded with treatment only for the LE. The patient passed away five years later due to the development of hepatic metastasis.

Patient BUM3: A 96-year-old Caucasian woman with no significant medical history was diagnosed with UM in her LE, 27 years after the enucleation of her RE due to UM at a different medical facility. Importantly, a melanocytic lesion had been present in her LE at the time of the right eye’s enucleation and was continuously monitored until the recent diagnosis of UM.

**Figure 3:**
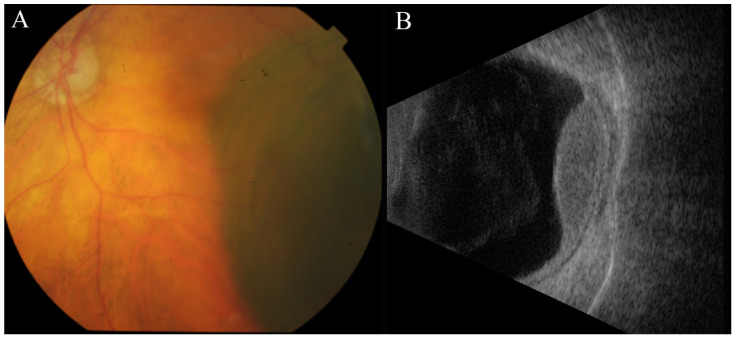
Imaging tests performed on the LE of patient BUM3, compatible with a diagnosis of BUM. (**A**) Fundus of the LE of patient BUM3 showing a choroidal pigmented mass in the temporal periphery. (**B**) Ocular ultrasound of LE showing a choroidal mass compatible with a UM measuring 4.5 mm in height and 15.3 mm in base.

Medical specialists retrieved a paraffin-embedded tumor sample from the enucleated RE to be included in a genomic study. Subsequent imaging tests revealed that the pigmented choroidal lesion in the temporal periphery of the LE had significantly enlarged over the previous eight months. This lesion exhibited prominent orange pigmentation and choroidal excavation, with a kappa angle evident on ultrasound—features consistent with UM (T2) (Figure 3). Despite the diagnosis, the patient opted against treatment for the UM in her LE. Regrettably, she passed away one year later.

**Figure 4:**
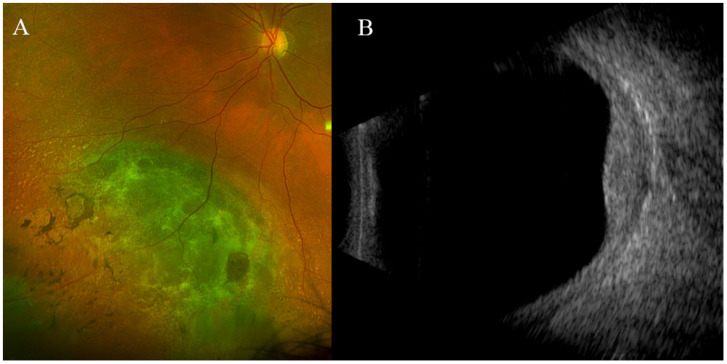
Imaging test results of the choroidal pigment mass under study in patient BUM4. (**A**) Eye fundus showing a mass with mixed pigmentation. (**B**) Ocular ultrasound of the LE displaying a choroidal mass compatible with uveal melanoma, measuring 3.6 mm in height and 14.3 mm in base.

Patient BUM4: A 72-year-old Caucasian woman was referred to our unit in 2016 for evaluation of a large melanocytic lesion in the LE. In 2001, she underwent an iridocyclectomy for uveal melanoma (fusiform B) in the RE. She remains under our care for a nodular choroidal mass in the LE, where a pigmented mass with significant growth is observed, which displays clinical and ultrasonographic features indicative of choroidal melanoma (Stage T2, with a thickness exceeding 3.5 mm) (Figure 4). The patient has opted not to undergo brachytherapy. In February 2019, she was diagnosed with primary infiltrating carcinoma of the left breast (hormone receptor-positive and HER2-negative). Currently, her liver enzyme levels are normal and there are no indications of metastatic disease, as confirmed by chest X-ray and abdominal ultrasound examinations.

Prioritized Variants and Candidate Gene Selection: This study first aimed to detect pathogenic or likely pathogenic variants in well-established UM and cancer-predisposing genes. None of the patients displayed pathogenic or likely pathogenic variants in the *BAP1* and *MBD4* genes or in other well-established cancer-predisposing genes (Supplementary Table S2).

Two rare heterozygous high-impact variants were identified in the Telomeric Repeat-Binding Factor 2-Interacting Protein 1 gene (*TERF2IP*) and the BCL2 Associated X, Apoptosis Regulator gene (*BAX*) in patients BUM4 and BUM3, respectively (Table 1). Patient BUM4 harbored a nonsense variant of unknown significance in the *TERF2IP* gene, p.(Arg364Ter), resulting in a truncated protein and disruption of the TERF2 binding domain. This variant had previously been associated with hereditary cutaneous malignant melanoma (CMM) [38,39]. No pathogenic or likely pathogenic variants were detected in the *TERF2IP* gene in the other three patients or in our in-house database of over three thousand sequenced exomes.

In the *BAX* gene, a likely pathogenic variant, *BAX*:c.243del p.(Ala82ProfsTer51), was detected in patient BUM3. This deletion shifts the protein reading frame and introduces a premature stop codon, probably causing protein truncation or the absence of protein expression. It has never been described before.

## 4. Discussion

Uveal melanoma is a type of cancer with few established risk factors. The calculated lifetime risk for BUM was estimated to be 1 in 50 million Caucasian people, indicating that one case of BUM is expected every 18 years in the USA [40]. Our study was predicated on the notion that the occurrence of a primary BUM, in the absence of other apparent clinical factors predisposing to it, suggests a genetic predisposition to this cancer type [1,8,40]. Identifying individuals at risk of developing UM and/or bilateral disease allows for the implementation of preventive cancer strategies, such as prophylactic surgeries or early interventions, to prevent or mitigate the progression of the disease. Therefore, focusing on obtaining useful germline genetic information can help predict the development of a tumor in the contralateral eye in a patient already affected by UM, or at least justify intensified ophthalmological examinations to detect this possibility.

Regarding published cases of BUM, so far only two studies have clearly established an association between BUM and the presence of germline mutations in a specific gene. The first describes the case of a woman with BUM (choroidal melanoma in her right eye and iris melanoma in her left eye) and basal cell carcinoma, who carried a pathogenic mutation in the *BAP1* gene, justifying a genetic diagnosis of BAP1-associated tumor predisposition syndrome [15]. The second study, published by Yu et al. a year later, describes the presence of germline *BAP1* mutations in two unrelated patients with primary BUM [7]. However, mutations in the *BAP1* gene do not account for all cases of BUM or UM with suspected genetic predisposition, suggesting that other genes with high or medium penetrance might also be involved in the occurrence of UM in patients without detectable alterations in the *BAP1* gene.

Consequently, we utilized an exome sequencing approach to identify novel rare UM predisposing variants potentially responsible for the development of bilateral disease. From the sequenced germline DNA of four unrelated Spanish patients with BUM, we selected the *TERF2IP* and *BAX* genes as candidates for further, more robust investigation (Table 1). To our knowledge, this represents the largest series of BUM cases analyzed simultaneously by extensive mass sequencing in the current literature.

Notably, none of the four BUM patients exhibited clinically significant variants in the *BAP1* gene. The frequency of germline *BAP1* mutations in UM has been estimated to range between 1.6 and 3.2% across different populations [41,42]. Although Aoude et al. [42] screened both unilateral and bilateral cases, it was not specified whether any bilateral cases harbored a *BAP1* mutation. As mentioned above, to date, to our knowledge, only three cases of BUM with confirmed presence of a *BAP1* germline pathogenic variant have been published [7,15]. The rarity of *BAP1* germline mutations in BUM patients, coupled with their low incidence in patients genetically predisposed to UM, suggests the potential involvement of other genes or high-risk factors in BUM predisposition.

Our study highlighted the Telomeric Repeat-Binding Factor 2-Interacting Protein 1 (*TERF2IP*) gene as particularly interesting. It encodes one of the most conserved components of the shelterin protein complex [43], which also includes proteins encoded by six other genes (*POT1*, *ACD*, *TPP1*, *TINT1*, *PIP1*, and *PTOP*) essential for telomere protection and DNA repair processes [43]. Following the discovery of predisposing germline mutations in the *POT1* gene, another shelterin complex component, for cutaneous malignant melanoma (CMM) [38,39,44], three families with germline variants in *TERF2IP*, including the nonsense mutation p.(R364Ter) and mutations co-segregating with cutaneous melanoma, were identified. These novel and rare germline variants in *TERF2IP* were statistically enriched in CMM probands compared to population controls [38,39]. Nathan et al. also reported a connection between germline truncating mutations in *POT1* and UM genetic predisposition [19]. Thus, we hypothesize that other dysfunctional genes involved in the shelterin complex, such as the *TERF2IP* gene, may also confer a risk for developing UM.

Truncating variants in *TERF2IP* are exceedingly rare in the gnomAD non-cancer population. Furthermore, the *TERF2IP*:p.(R364Ter) variant has not been previously detected in our in-house genetic repository, which contains over three thousand sequenced exomes. Despite the gnomAD pLI (probability of loss-of-function intolerance) does not classify *TERF2IP* as a loss-of-function intolerant gene, a gene damage index assessment study places *TERF2IP* among the top 20% of human genes regarding mutation intolerance [45], and ranks within the top 10% for “functional indispensability” genes [46]. Additionally, the *TERF2IP* protein is crucial for connecting to the shelterin complex, engaging with TERF2 through its C-terminal end to form a critical complex. This interaction is essential for inhibiting the homology-directed repair of double-stranded chromosomal breaks at telomeres. Aoude et al. have highlighted that the mutation *TERF2IP*:p.(R364Ter) leads to an early truncation of the protein, eliminating 36 amino acids from the C-terminal end. This alteration disrupts the domain that binds to TERF2, potentially leading to a failure in its connection with the shelterin complex and thus hindering its protective role at telomeres [38].

Families with germline mutations in *TERF2IP* are associated with early onset of CMM and a predisposition to a broad spectrum of primary cancers, including breast, lung, cervix, colon, bowel, ovary, and B-cell lymphoma, suggesting mutations in this gene may predispose to a wider range of cancers than just CMM [39]. Yet, its link to UM development remains unexplored. In our study, the identified truncating variant in *TERF2IP* occurred in a patient who also developed primary breast cancer (BUM4), hinting at a possible tumor predisposition syndrome associated with the *TERF2IP* gene, similar to the one described for the *POT1* gene [38]. However, this evidence is not sufficient to definitively claim *TERF2IP* as a candidate for UM susceptibility but suggests promising directions for future research.

Another gene of interest was *BAX*, which encodes a pro-apoptotic protein essential for cell death in response to various stimuli that are directly activated by p53 [47,48]. Mutations in *BAX* have been suggested to confer cancer predisposition, leading to a phenotype resembling Li-Fraumeni syndrome [49]. However, the role of germline alterations in the *BAX* gene in cancer, especially in *TP53* wild-type Li-Fraumeni families, remains debated [49]. A germline deletion in the *BAX* gene was identified in a patient with both cutaneous melanoma and a gastrointestinal stromal tumor [50], and somatic BAX protein alterations were observed in various cancers [51]. Further research is needed to clarify whether *BAX* alterations constitute a cancer risk factor, particularly in UM-enriched patient or family populations.

It is important to emphasize that this study is mainly exploratory and descriptive, highlighting potential genes for BUM/UM susceptibility but not providing conclusive evidence. The small number of BUM patients analyzed introduces significant uncertainty to statistical outcomes. Furthermore, an additional limitation of our study was the inability to perform sequencing analysis on the tumor samples from the patients, which would have aided significantly in understanding, prioritizing, and definitively selecting candidate genes. This is particularly crucial because another marker of germline variant causality in cancer involves identifying a second mutational event in the tumor sample within the same gene where a pathogenic variant was found at the germline level. Given these challenges, the establishment of international collaborative groups would be extremely beneficial for conducting studies of this nature, especially for exceedingly rare conditions like the one discussed in this study. Nevertheless, conducting such an extensive genomic study on an extremely rare disease is noteworthy and, to our opinion, deserves publication.

Moreover, the bilateral development of UM does not imply a uniform genetic cause. In fact, the potential oligogenic nature of UM is supported by the absence of pathogenic variants in *BAP1* and other UM predisposition genes (*MDB4*), as well as the lack of novel recurrent mutated genes in our patients. Additionally, we believe that other risk factors and the interaction of multiple genetic variants with environmental factors may also contribute to BUM development, suggesting a multifactorial etiology for this disease. To address these limitations, larger studies are necessary.

## 5. Conclusions

Primary bilateral uveal melanoma is an exceptionally rare condition, which in some instances can be attributed to germline mutations in the *BAP1* gene. However, not all cases of this disease can be explained by mutations in this gene alone. In fact, the lack of *BAP1* germline mutations among the four BUM patients we studied underscores the genetic diversity underlying UM predisposition. Our research proposes two genes, *TERF2IP* and *BAX*, as potential candidates for further detailed examination in the context of UM predisposition. While the evidence for the variants in these genes is currently limited and they may represent sporadic occurrences, we propose that our findings lay a significant groundwork for subsequent research into UM predisposition. Future investigations should aim to validate or refute the connection between these variants and UM, using their own datasets for comprehensive analysis. Clarifying potential germline predispositions to cancer is essential for developing effective cancer prevention strategies. Consequently, it is important for patients who are clinically suspected of having a genetic predisposition to UM, including those with BUM, to undergo genetic testing. This testing should assess not only predisposition to UM but also other cancer predisposition syndromes. The pursuit of such studies necessitates collaborative efforts across multidisciplinary research teams, especially for examining ultra-rare conditions like BUM, which are likely to exhibit both molecular and clinical heterogeneity. Moreover, integrating complementary functional studies, such as CRISPR assays, could enhance the effectiveness of screening for genes that confer susceptibility to UM.

## Figures and Tables

**Table 1:** Description of the selected variants for a more in-depth investigation in BUM patients. Two rare, high-impact variants were found in two (BUM3 and BUM4) of the four patients studied. No variants in these genes were found in either of the other two patients (BUM1 and BUM2). Reference sequences: ^a^ *BAX*(NM_138761.4); ^b^ *TERF2IP*(NM_018975.4)//* LP: likely pathogenic; VUS: variant of uncertain significance//ACMG: American College of Medical Genetics and Genomics.

Patient	Gene ^ab^	Variant Description	Protein Effect	gnomAD Freq.	ACMG Classification *	ACMG Criteria
BUM3	*BAX*	c.243del p.(Ala82ProfsTer51)	premature stop codon	0	LP	PVS1, PM2
BUM4	*TERF2IP*	c.1090C>T p.(R364Ter)	premature stop codon	0.0000199	VUS	PVS1_strong, PM2

## Data Availability

Data are contained within the article.

## Supplementary Materials

The following supporting information can be downloaded at: https://www.mdpi.com/article/10.3390/jcm13113035/s1, Table S1: Summary table of the studies published to date about primary bilateral uveal melanoma (adapted and updated from Scott et al. 2018 [8]). SC: Series of cases. CR: case report. LE: left eye. RE: right eye; Table S2: Selection of well-known candidate genes predisposing to uveal melanoma and other types of cancer for the analysis of rare variants in the four BUM patients.
