# Supplementary material for: Bilateral Uveal Melanoma: An Insight into Genetic Predisposition in Four New Unrelated Patients and Review of Published Cases
Source: J Clin Med. 2024 May 22;13(11):3035. doi: 10.3390/jcm13113035 (PMC11172988; doi:10.3390/jcm13113035)
Supplement: Supplementary file 1 [file jcm-13-03035-s001.zip › jcm-2987444-supplementary.pdf]

Supplementary Table S1. Summary table of the studies published to date about primary bilateral uveal melanoma (adapted and updated from Scott et al. 2018). SC: Series of cases. CR: case report. LE: left eye. RE: right eye.

Title of the publication	Year	Authors	Study design	Number of cases	Tumor features	Treatment method	Time interval of diagnosis	germline genetic finding
A case of malignant melanoma of the choroid successfully treated by radom seeds.	1949	Stallard et al.	CR	1	-	Radom seeds.	-	-
Simultaneous bilateral primary ocular malignant melanoma; report of a case.	1950	Cordes et al.	CR	1	RE: iris melanoma (spindle cell A and B).<br>LE: choroidal melanoma (fascicular type).	RE: iridectomy.<br>LE: enucleation.	simultaneous	-
Bilateral melanoma of the choroid associated with leukemia and meningioma.	1959	Wiesinger et al.	CR	1	Bilateral choroidal melanoma.	-	-	-
Bilateral choroidal melanomas. Case report and incidence.	1977	Shammas&Wazke.	CR	1	Bilateral choroidal melanoma.	-	-	-
Bilateral malignant choroidal melanomas.	1980	Lubin et al.	SC	3	Bilateral choroidal melanoma.	-	-	-
Treatment of bilateral choroidal malignant melanoma.	1981	Lommatzsch et al.	CR	1	Bilateral choroidal melanoma.	ruthenium plaque for both eyes + enucleation of one eye.	simultaneous	-
Bilateral primary choroidal melanoma.	1984	Migdal et al.	CR	1	RE: choroidal melanoma (mixed spindle B + epithelioid types).<br>LE: choroidal melanoma (spindle cells).	RE: iridectomy and anterior vitrectomy.<br>LE: enucleation.	2 years	-
Histopathology of uveal melanomas treated with charged particle radiation.	1987	Crawford et al.	CR	1	Bilateral choroidal melanoma.	radiation	-	-
Bilateral uveal melanomas.	1987	Gailloud et al.	SC	3	Bilateral choroidal melanoma (n = 3).	-	-	-
Two cases of primary bilateral malignant melanoma of the choroid.	1988	Seregard et al.	SC	2	**Case 1:** bilateral choroidal melanoma (RE: epithelioid cells).<br>**Case 2:** bilateral choroidal melanomas (spindle B cell type).	**Case 1:**<br>RE: enucleation.<br>LE: ruthenium plaque.<br>**Case 2:** enucleation for both eyes.	**Case 1:** simultaneous.<br>**Case 2:** simultaneous.	-

Bilateral ciliary body melanomas.	1989	Waterhouse et al.	CR	1	Bilateral ciliary body melanomas (B spindle cell type).	iridocyclectomy	6 months	-
Simultaneous bilateral primary choroidal melanoma.	1989	Eide et al.	CR	1	RE: choroidal melanoma<br>LE: choroidal melanoma (mixed cell type).	RE: radiation.<br>LE: enucleation.	simultaneous	-
Bilateral uveal melanoma presenting simultaneously.	1994	Omulecki et al.	CR	1	RE: ciliary body melanoma (A/B spindle-cell type).<br>LE: choroidal melanoma (A/B spindle-cell type).	RE: local tumor excision.<br>LE: enucleation.	simultaneous	-
Simultaneous bilateral primary choroidal melanoma.	1996	Ascaso et al.	CR	1	RE: choroidal melanoma (mixed cell type).<br>LE: choroidal melanoma	RE: enucleation.<br>LE: Laser photocoagulation and plaque of ruthenium-106.	simultaneous	-
Bilateral primary uveal melanoma. Bad luck or bad genes?	1996	Singh et al.	SC	8	-	-	-	-
Bilateral malignant melanoma of the choroid in the United States.	1998	Ruiz et al.	CR	1	Bilateral choroidal melanoma.	-	simultaneous	
Sequential development of bilateral primary choroidal melanoma.	2000	Kiralti et al.	CR	1	Bilateral choroidal melanoma.	RE: radioactive plaque.<br>LE: thermotherapy.	20 months	-
Bilateral uveal melanoma: a series of four cases.	2003	Hadden et al.	SC	4	**Case 1:**<br>RE: choroidal melanoma.<br>LE: choroidal melanoma.<br>**Case 2:**<br>RE: choroidal melanoma.<br>LE: diffuse choroidal melanoma.<br>**Case 3:**<br>bilateral choroidal tumors, spindle B cell type.<br>**Case 4:** bilateral choroidal melanomas.	**Case 1:**<br>RE: krypton laser + endoresection.<br>LE: Proton beam radiotherapy.<br>**Case 2:**<br>bilateral plaque radiotherapy.<br>**Case 3:**<br>bilateral proton beam radiotherapy.<br>**Case 4:**<br>RE: radiotherapy<br>LE: radiotherapy	**Case 1:** 9 years<br>**Case 2:** simultaneous<br>**Case 3:** simultaneous<br>**Case 4:** 3 years	-
Bilateral primary choroidal.	2006	Futura et al.	SC	3	**Case 1:**<br>RE: choroidal melanoma<br>LE: choroidal melanoma<br>**Case 2:**	bilateral plaque radiotherapy	**Case 1:** 13 years<br>**Case 2:** 18 years<br>**Case 3:** 2 years	-

					RE: choroidal melanoma<br>LE: amelanotic choroidal melanoma<br>**Case 3:**<br>RE: choroidal melanoma<br>LE: choroidal melanoma			
Simultaneous appearance of acute myeloid leukemia in a patient with bilateral primary uveal melanoma.	2006	Buda et al.	CR	1	-	-	6 years	-
Bilateral uveal melanoma in an arc welder.	2011	Turaka et al.	CR	1	RE: choroidal melanoma.<br>LE: iridociliary melanoma (spindle B-cell).	RE: radiotherapy.<br>LE: radiotherapy.	4 years	-
Prevalence of ocular and oculodermal melanocytosis in Spanish population with uveal melanoma.	2012	Carreno et al.	CR	1	Bilateral choroidal melanoma.	brachytherapy.	simultaneous	-
Bilateral choroidal melanoma-case analysis and literature review.	2015	Kowal et al.	SC	5	**Case 1:**<br>RE: choroidal melanoma (mixed cell type).<br>LE: choroidal melanoma.<br>**Case 2:**<br>RE: choroidal melanoma.<br>LE: choroidal melanoma.<br>**Case 3:**<br>RE: choroidal melanoma (mixed cell type).<br>LE: choroidal melanoma.<br>**Case 4:**<br>RE: choroidal melanoma.<br>LE: iris melanoma (mixed cell type).<br>**Case 5:**<br>RE: choroidal melanoma.<br>LE: choroidal melanoma.	**Case 1:**<br>RE: brachytherapy + enucleation.<br>LE: brachytherapy.<br>**Case 2:**<br>RE: enucleation.<br>LE: brachytherapy.<br>**Case 3:**<br>RE: brachytherapy + thermotherapy + enucleation.<br>LE: Ru-106 brachytherapy.<br>**Case 4:**<br>RE: brachytherapy.<br>LE: iris tumor resection.<br>**Case 5:** proton beam radiotherapy.	**Case 1:** 32 years.<br>**Case 2:** 21 years.<br>**Case 3:** 2 years.<br>**Case 4:** simultaneous.<br>**Case 5:** simultaneous.	-
Bilateral uveal melanomas with different gene expression detected with 7 years interval.	2016	Eide et al.	CR	1	RE: amelanotic juxtapupillary tumor (mixed cell type).<br>LE: melanotic choroidal melanoma (spindle cell type)	RE: enucleation.<br>LE: plaque.	7 years	-
Cleveland University Hospital Pathology Database	1996-2016	Scott et al.2018	CR	1	not specified	not specified	not specified	not specified

Surveillance, Epidemiology and End-Results (SEER)-18 Database	1973-2013	Scott et al. 2018	SC	5	not specified	not specified	not specified	not specified
Basal cell carcinomas developing independently from BAP1-tumor predisposition syndrome in a patient with bilateral uveal melanoma: Diagnostic challenges to identify patients with BAP1-TPDS	2019	Melzer et al.	CR	1	RE: choroidal melanoma.<br>LE: iris melanoma.	LE: excision of the tumor.<br>RE: not specified.	not specified	*BAP1* germline mutation
BAP1 Germline Mutation Associated with Bilateral Primary Uveal Melanoma.	2020	Yu MD et al.	SC	2	**Case 1:**<br>RE: choroidal melanoma.<br>LE: choroidal melanoma.<br>**Case 2:**<br>RE: diffuse iris melanoma (epithelioid cell).<br>LE: ciliary body melanoma	**Case 1:**<br>RE: enucleation.<br>LE: radiotherapy.<br>**Case 2:**<br>RE: radiotherapy + enucleation.<br>LE: radiotherapy.	**Case 1:** 4 years<br>**Case 2:** 4 years.	*BAP1* germline mutations in two unrelated patients
Whole Exome Sequencing Identifies Candidate Genes Associated with Hereditary Predisposition to Uveal Melanoma	2020	Abdel-Rhaman MH.	SC	1	not specified	not specified	not specified	not specified
Multiple Uveal Melanoma	2020	Wajiha J Kheir et al.	CR	1	bilateral choroidal melanoma	brachytherapy	simultaneous	*BRCA2*

Supplementary Table S2: Selection of well-known candidate genes predisposing to uveal melanoma and other types of cancer for the analysis of rare variants in the four BUM patients.

Gene category	Gene symbol	Gene name	Cancer associated syndrome(s)/associated condition	Associated tumor types
Uveal melanoma predisposing genes	*BAP1**	BRCA1 associated protein-1 (ubiquitin carboxy-terminal hydrolase)	BAP1-Tumor Predisposition Syndrome	Melanoma (cutaneous, uveal)<br>Mesothelioma<br>Meningioma<br>Renal carcinoma<br>Lung cancer (adenocarcinoma)
	*MBD4***	*Methyl-CpG Binding Domain 4, DNA Glycosylase*	Progressive familial intrahepatic cholestasis	Uveal melanoma
Other well-Known Cancer Predisposing genes and DNA repair involved genes***	*ABCB11*	ATP-binding cassette, sub-family B (MDR/TAP), member 11	Progressive familial intrahepatic cholestasis	Hepatocellular carcinoma<br>Cholangiocarcinoma
	ALK	anaplastic lymphoma receptor tyrosine kinase		Neuroblastoma
	APC	adenomatous polyposis coli	Familial adenomatous polyposis (FAP)	Colorectal cancer<br>Hepatoblastoma<br>Desmoid tumor
	ATM	ataxia telangiectasia mutated	Ataxia-Telangiectasia (biallelic mutations)	Biallelic mutations:<br>Lymphoid hematological malignancy (leukemia, lymphoma)<br>Monoallelic mutations:<br>Breast cancer
	AXIN2	axin 2	oligodentia-colorectal cancer syndrome	Colorectal cancer
	BLM	Bloom syndrome, RecQ helicase-like	Bloom syndrome	Lymphoma and ALL hematological malignancy<br>Myeloid hematological malignancy<br>Squamous cell carcinoma, scc<br>gastic, colorectal cancers
	BMPR1A	bone morphogenetic protein receptor, type IA	Juvenile polyposis syndrome	Colorectal cancer, gastric cancer, hamartoma
	BRCA1	breast cancer 1, early onset	Hereditary breast-ovarian cancer	Breast cancer<br>Ovarian cancer

	BRCA2	breast cancer 2, early onset	Hereditary breast-ovarian cancer<br>Fanconi anaemia (D1) (biallelic mutations)	<u>Biallelic mutations:</u><br>Myeloid hematological malignancy (<br>Medulloblastoma<br>Wilms tumor<br><u>Monoallelic mutations:</u><br>Breast cancer<br>Ovarian cancer<br>Prostate cancer<br>Pancreas cancer
	BRIP1	BRCA1 interacting protein C-terminal helicase 1	Fanconi anaemia (J) (biallelic mutations)	<u>Biallelic mutations:</u><br>Myeloid hematological malignancy<br>Squamous cell carcinoma (head and neck, esophagus, genital tract)<br><u>Monoallelic mutations:</u><br>Breast cancer<br>Ovarian cancer
	BUB1B	budding uninhibited by benzimidazoles 1 homolog beta (yeast)	Mosaic variegated aneuploidy Syndrome	Wilms Tumor<br>Rhabdomyosarcoma<br>Myeloid hematological malignancy
	CBL	Cbl proto-oncogene, E3 ubiquitin protein ligase	Noonan syndrome	JMML
	CDC73	cell division cycle 73, Paf1/RNA polymerase II complex component, homolog (S. cerevisiae)	Hyperparathyroidism-jaw tumor syndrome	Parathyroid cancer<br>Ossifying fibroma (bone)
	CDH1	cadherin 1, type 1, E-cadherin (epithelial)	Hereditary diffuse gastric cancer	Breast cancer (lobular)<br>Gastric cancer (diffuse)
	CDK4	cyclin-dependent kinase 4		Melanoma
	CDKN1B	cyclin-dependent kinase inhibitor 1B (p27, Kip1)		Thyroid cancer,<br>Pituitary adenoma

	CDKN2A	cyclin-dependent kinase inhibitor 2A		Melanoma [p16 and p14ARF]<br>Pancreas cancer [p16]<br>Astrocytoma [p14ARF]
	CEBPA	CCAAT/enhancer binding protein (C/EBP), alpha		Myeloid hematological malignancy
	CHEK2	checkpoint kinase 2		Breast cancer
	COL7A1	collagen, type VII, alpha 1	Epidermolysis bullosa	Squamous cell carcinmona (skin)
	CYLD	cylindromatosis (turban tumor syndrome)	Brooke-Spiegler syndrome	Cylindroma<br>spiroadenocarcinoma<br>Basal cell carcinoma
	DDB2	damage-specific DNA binding protein 2, 48kDa	Xeroderma Pigmentosum (E)	Basal cell carcinoma<br>Squamous cell carcinoma<br>Melanoma
	DICER1	dicer 1, ribonuclease type III	DICER1 syndrome	Pleuropulmonary blastoma<br>Cystic nephroma<br>Ovarian sex cord tumor
	DIS3L2	DIS3 mitotic control homolog (S. cerevisiae)-like 2	Perlman syndrome	Wilms tumor
	DKC1	dyskeratosis congenita 1, dyskerin	Dyskeratosis congenita	acute myeloid leukemia<br>Squamous cell carcinoma (head + neck, anorectal)
	DOCK8	dedicator of cytokinesis 8	HyperIgE syndrome	Squamous cell carcinoma<br>Lymphoma
	EGFR	epidermal growth factor receptor		Non-small cell lung cancer
	ELANE	elastase, neutrophil expressed	Severe congenital neutropenia	Leukemia
	ERCC2	excision repair cross-complementing rodent repair deficiency, complementation group 2	Xeroderma pigmentosum (D)	Basal cell carcinoma<br>Squamous cell carcinoma<br>Melanoma

	ERCC3	excision repair cross-complementing rodent repair deficiency, complementation group 3	Xeroderma pigmentosum (B)	Basal cell carcinoma<br>Squamous cell carcinoma<br>Melanoma
	ERCC4	excision repair cross-complementing rodent repair deficiency, complementation group 4	Xeroderma pigmentosum (F)<br>Fanconi anaemia (Q)	Basal cell carcinoma<br>Squamous cell carcinoma<br>Melanoma
	ERCC5	excision repair cross-complementing rodent repair deficiency, complementation group 5	Xeroderma pigmentosum (G)	Basal cell carcinoma<br>Squamous cell carcinoma<br>Melanoma
	EXT1	exostosin 1		Chondrosarcoma
	EXT2	exostosin 2		Chondrosarcoma
	FAH	fumarylacetoacetate hydrolase (fumarylacetoacetase)	Tyrosinemia	Hepatocellular carcinoma
	FANCA	Fanconi anemia, complementation group A	Fanconi anaemia (A)	Myeloid hematological malignancy<br>Squamous cell carcinoma (head and neck, esophagus, genital tract)
	FANCC	Fanconi anemia, complementation group C	Fanconi anaemia (C)	Myeloid hematological malignancy<br>Squamous cell carcinoma (head and neck, esophagus, genital tract)
	FANCG	Fanconi anemia, complementation group G	Fanconi anaemia (G)	Myeloid hematological malignancy<br>Squamous cell carcinoma (head and neck, esophagus, genital tract)
	FH	fumarate hydratase	Hereditary leiomyomatosis and renal cell cancer (HLRCC)	Renal cell cancer<br>Leiomyosarcoma (uterus)
	FLCN	folliculin	Birt-Hogg-Dube syndrome	Renal cell cancer<br>Oncocytoma
	GATA2	GATA binding protein 2	Emberger MonoMAC syndrome	Myeloid hematological malignancy

	GBA	glucosidase, beta, acid	Gauchers type 1	Myeloma<br>Lymphoma<br>Hepatocellular carcinoma
	GJB2	gap junction protein, beta 2, 26kDa	Keratosis-icthyosis-deafness syndrome (KID)	Squamous cell carcinoma
	GPC3	glypican 3	Simpson-Golabi-Behmel syndrome	Wilms tumor<br>Hepatoblastoma, hepatocellular carcinoma<br>Neuroblastoma<br>Gonadoblastoma
	HFE	hemochromatosis	Haemochromatosis	Hepatocellular carcinoma<br>Cholangiocarcinoma
	HMBS	hydroxymethylbilane synthase	Porphyria (AI)	hepatocellular carcinoma
	HRAS	v-Ha-ras Harvey rat sarcoma viral oncogene homolog	Costello syndrome	Rhabdomyosarcoma<br>Neuroblastoma<br>Transitional cell carcinoma (bladder)
	ITK	IL2-inducible T-cell kinase	Lymphoproliferative syndrome 1	Hodgkins lymphoma
	KIT	v-kit Hardy-Zuckerman 4 feline sarcoma viral oncogene homolog		Gastro-Intestinal Stromal Tumor
	MAX	MYC associated factor X	Familial paraganglioma-pheochromocytoma syndrome	Paraganglioma<br>Pheochromocytoma
	MEN1	multiple endocrine neoplasia I	Multiple endocrine neoplasia Type 1	Parathyroid, pituitary adenoma<br>Neuroendocrine tumor<br>Carcinoid tumor<br>Adrenocortical carcinoma
	MET	met proto-oncogene (hepatocyte growth factor receptor)		Renal cell cancer (papillary carcinoma)

	MLH1	mutL homolog 1, colon cancer, nonpolyposis type 2 (E. coli)	MMR deficiency syndrome (biallelic mutations)<br>Lynch syndrome / Hereditary Non-Polyposis Colon Cancer (monoallelic mutations)	<u>Biallelic mutations:</u><br>Brain tumors<br>Hematological malignancy<br>Embryonal tumors<br><u>Monoallelic mutations:</u><br>Colorectal cancer<br>Endometrial cancer<br>Ovarian cancer
	MSH2	mutS homolog 2, colon cancer, nonpolyposis type 1 (E. coli)	MMR deficiency syndrome (biallelic mutations)<br>Lynch syndrome / Hereditary Non-Polyposis Colon Cancer (monoallelic mutations)	<u>Biallelic mutations:</u><br>Brain tumors<br>Hematological malignancy<br>Embryonal tumors<br><u>Monoallelic mutations:</u><br>Colorectal cancer<br>Endometrial cancer<br>Ovarian cancer<br>Sebaceous adenoma, carcinoma, epithelioma
	MSH6	mutS homolog 6 (E. coli)	MMR deficiency syndrome (biallelic mutations)<br>Lynch syndrome / Hereditary Non-Polyposis Colon Cancer (monoallelic mutations)	<u>Biallelic mutations:</u><br>Brain tumors<br>Hematological malignancy<br>Embryonal tumors<br><u>Monoallelic mutations:</u><br>Colorectal cancer<br>Endometrial cancer<br>Ovarian cancer

	MTAP	methylthioadenosine phosphorylase	Diaphyseal medullary stenosis with malignant fibrous histiocytoma (DMS-MFH)	malignant fibrous histiocytoma (sarcoma)
	MUTYH	mutY homolog (E. coli)		Colorectal cancer
	NBN	nibrin	Nijmegen breakage syndrome	Lymphoma<br>Medulloblastoma<br>Glioma<br>Rhabdomyosarcoma
	NF1	neurofibromin 1	Neurofibromatosis type 1	Glioma<br>Malignant peripheral nerve sheath tumor
	NF2	neurofibromin 2 (merlin)	Neurofibromatosis type 2	Vestibular schwannoma<br>Meningioma<br>Ependymoma
	PALB2	partner and localizer of BRCA2	Fanconi anaemia (N) (biallelic mutations)	<u>Biallelic mutations:</u><br>Myeloid hematological malignancy<br>Medulloblastoma<br>Neuroblastoma<br>Wilms tumor<br><u>Monoallelic mutations:</u><br>Breast cancer<br>Pancreas cancer
	PDGFRA	platelet-derived growth factor receptor, alpha polypeptide		Gastro-Intestinal Stromal Tumor
	PHOX2B	paired-like homeobox 2b		Neuroblastoma

	PMS2	PMS2 postmeiotic segregation increased 2 (S. cerevisiae)	MMR deficiency syndrome (biallelic mutations)<br>Lynch syndrome / Hereditary Non-Polyposis Colon Cancer (monoallelic mutations)	<u>Biallelic mutations:</u><br>Brain tumors<br>Hematological malignancy<br>Supratentorial primitive neuroectodermal tumors<br><u>Monoallelic mutations:</u><br>Colorectal cancer<br>Endometrial cancer<br>Ovarian cancer
	POLD1	polymerase (DNA directed), delta 1, catalytic subunit	PPAP (polymerase proofreading associated polyposis)	Colorectal cancer<br>Endometrial cancer
	POLE	polymerase (DNA directed), epsilon, catalytic subunit	PPAP (polymerase proofreading associated polyposis)	Colorectal cancer
	POLH	polymerase (DNA directed), eta	Xeroderma pigmentosa V	Squamous cell cancer (skin)
	PRKAR1A	protein kinase, cAMP-dependent, regulatory, type I, alpha	Carney complex	Myxoma (cardiac/cutaneous/breast)<br>Thyroid cancer<br>Sex cord-stromal tumor
	PRSS1	protease, serine, 1 (trypsin 1)		Pancreatic cancer
	PTCH1	patched 1	Nevoid basal cell carcinoma syndrome<br>Gorlin Syndrome	Basal cell carcinoma<br>Medulloblastoma
	PTEN	phosphatase and tensin homolog	Cowden Syndrome<br>PTEN hamartoma tumor syndrome	Breast cancer<br>Thyroid cancer<br>Endometrial cancer
	PTPN11	protein tyrosine phosphatase, non-receptor type 11	Noonan syndrome	JMML<br>neuroblastoma

	RAD51C	RAD51 homolog C (S. cerevisiae)	Fanconi anaemia (O) (biallelic mutations)	Monoallelic mutations:<br>Ovarian cancer
	RAD51D	RAD51 homolog D (S. cerevisiae)		Ovarian cancer
	RB1	retinoblastoma 1		Retinoblastoma<br>Pinealoma<br>Sarcoma<br>Melanoma
	RECQL4	RecQ protein-like 4	Rothmund-Thompson syndrome	Osteosarcoma<br>Basal cell carcinoma<br>Squamous cell carcinoma
	RET	ret proto-oncogene	Multiple endocrine neoplasia 2A/2B<br>Familial medullary thyroid carcinoma	Medullary thyroid cancer<br>Pheochromocytoma
	RHBDF2	rhomboid 5 homolog 2 (Drosophila)		Esophageal cancer
	RMRP	RNA component of mitochondrial RNA processing endoribonuclease	Cartilage-hair hypoplasia syndrome	Non-hodgkin lymphoma<br>Squamous carcinoma (bcc)<br>Leukemia
	RUNX1	runt-related transcription factor 1		Myeloid hematological malignancy (leukemia)
	SBDS	Shwachman-Bodian-Diamond syndrome	Schwachman-Diamond syndrome	Myeloid hematological malignancy
	SDHA	succinate dehydrogenase complex, subunit A, flavoprotein (Fp)	Carney-Stratakis syndrome	Paraganglioma<br>Pheochromocytoma<br>Gastrointestinal stromal tumor (GIST)
	SDHAF2	succinate dehydrogenase complex assembly factor 2	Familial paraganglioma-pheochromocytoma syndrome	Paraganglioma<br>Pheochromocytoma
	SDHB	succinate dehydrogenase complex, subunit B, iron sulfur (Ip)	Familial paraganglioma-pheochromocytoma syndrome	Paraganglioma<br>Pheochromocytoma<br>Renal cell cancer

	SDHC	succinate dehydrogenase complex, subunit C, integral membrane protein, 15kDa	Familial paraganglioma-pheochromocytoma syndrome	Paraganglioma<br>Pheochromocytoma<br>Gastrointestinal stromal tumor (GIST)
	SDHD	succinate dehydrogenase complex, subunit D, integral membrane protein	Familial paraganglioma-pheochromocytoma syndrome	Paraganglioma<br>Pheochromocytoma<br>Gastrointestinal stromal tumor (GIST)
	SERPINA1	serpin peptidase inhibitor, clade A (alpha-1 antiproteinase, antitrypsin), member 1	Alpha1 antitrypsin defiency	Hepatocellular carcinoma
	SH2D1A	SH2 domain containing 1A	Lymphoproliferative disease	Lymphoma
	SLC25A13	solute carrier family 25 (aspartate/glutamate carrier), member 13	Citrullinaemia	Hepatocellular carcinoma
	SMAD4	SMAD family member 4	Juvenile polyposis syndrome	Colorectal cancer
	SMARCA4	SWI/SNF related, matrix associated, actin dependent regulator of chromatin, subfamily a, member 4	Rhabdoid predisposition syndrome	Rhabdoid tumor
	SMARCB1	SWI/SNF related, matrix associated, actin dependent regulator of chromatin, subfamily b, member 1	Rhabdoid predisposition syndrome	Rhabdoid tumor (renal, extra-renal)<br>Central primitive neuroectodermal tumor
	SMARCE1	SWI/SNF related, matrix associated, actin dependent regulator of chromatin, subfamily e, member 1		Mengingioma
	SOS1	son of sevenless homolog 1 (Drosophila)	Noonan syndrome	Rhabdomyosarcoma
	SRY	sex determining region Y		Gonadoblastoma
	STAT3	signal transducer and activator of transcription 3 (acute-phase response factor)	Hyper-immunoglobulin E syndrome	Lymphoma

	STK11	serine/threonine kinase 11	Peutz-Jeghers syndrome	Colorectal cancer<br>Gastric cancer<br>Breast cancer<br>Sex cord-stromal tumor
	SUFU	suppressor of fused homolog (Drosophila)		Medulloblastoma, meningioma
	TERT	telomerase reverse transcriptase	Dyskeratosis congenita	acute myeloid leukemia<br>Squamous cell carcinoma (head + neck, anorectal)<br>Melanoma
	TGFBR1	transforming growth factor, beta receptor 1	Multiple self-healing squamous epithelioma (MSSE)<br>Ferguson-Smith syndrome	Squamous cell carcinoma (skin)
	TMEM127	transmembrane protein 127		Pheochromocytoma
	TNFRSF6 (FAS)	transforming growth factor, beta receptor 1	Autoimmunie lymphoproliferative syndrome	Lymphoma
	TP53	tumor protein p53	Li-Fraumeni syndrome	Breast cancer<br>Sarcoma<br>Adrenocortical carcinoma<br>Astrocytoma
	TRIM37	tripartite motif containing 37	Mulibrey-nanism	Wilms tumor

	TSC1	tuberous sclerosis 1	Tuberous sclerosis 1	Renal cell cancer, angiomyolipoma<br>Subependymal giant cell astrocytoma<br>Rhabdomyoma (cardiac)
	TSC2	tuberous sclerosis 2	Tuberous sclerosis 2	Renal cell cancer, angiomyolipoma<br>Subependymal giant cell astrocytoma<br>Rhabdomyoma (cardiac)
	UROD	uroporphyrinogen decarboxylase	Porphyria (cutanea tarda)	hepatocellular carcinoma
	VHL	von Hippel-Lindau tumor suppressor, E3 ubiquitin protein ligase	Von Hippel-Lindau syndrome	Renal cell cancer<br>Pheochromocytoma<br>Neuroendocrine tumor (pancreas)<br>Hemangioblastoma (central nervous system, retina)
	WAS	Wiskott-Aldrich syndrome	Wiskott-Aldrich syndrome<br>WAS-related syndrome	Lymphoma
	WRN	Werner syndrome, RecQ helicase-like	Werner syndrome	Sarcoma<br>Melanoma<br>Thyroid cancer
	WT1	Wilms tumor 1	WAGR syndrome<br>Denys-Drash syndrome<br>Frasier syndrome	Wilms tumor<br>Gonadoblastoma
	XPA	xeroderma pigmentosum, complementation group A	Xeroderma pigmentosum (A)	Basal cell carcinoma<br>Squamous cell carcinoma<br>Melanoma

	XPC	xeroderma pigmentosum, complementation group C	Xeroderma pigmentosum (C)	Basal cell carcinoma<br>Squamous cell carcinoma<br>Melanoma

*****Abdel-Rahman MH, Pilarski R, Cebulla CM, Massengill JB, Christopher BN, Boru G, Hovland P, Davidorf FH. Germline BAP1 mutation predisposes to uveal melanoma, lung adenocarcinoma, meningioma, and other cancers. J Med Genet. 2011 Dec;48(12):856-9. doi: 10.1136/jmedgenet-2011-100156. Epub 2011 Sep 22. PMID: 21941004; PMCID: PMC3825099.
******Derrien AC, Rodrigues M, Eeckhoutte A, Dayot S, Houy A, Mobuchon L, Gardrat S, Lequin D, Ballet S, Pierron G, Alsafadi S, Mariani O, El-Marjou A, Matet A, Colas C, Cassoux N, Stern MH. Germline MBD4 Mutations and Predisposition to Uveal Melanoma. J Natl Cancer Inst. 2021 Jan 4;113(1):80-87. doi: 10.1093/jnci/djaa047. PMID: 32239153; PMCID: PMC7781447.
*******Rahman, N. Realizing the promise of cancer predisposition genes. Nature 505, 302–308 (2014). https://doi.org/10.1038/nature12981.
***Ruttan CC, Glickman BW. Coding variants in human double-strand break DNA repair genes. Mutat Res. 2002 Nov 30;509(1-2):175-200. doi: 10.1016/s0027-5107(02)00218-x. PMID: 12427538.
